# The clinical validity of miR-126 as a prognostic marker in epithelial ovarian cancer

**DOI:** 10.1097/MD.0000000000033085

**Published:** 2023-03-03

**Authors:** Lin Liu, Qing Han, Jing Cai, Man Xiao, Da Huang, Jin Cao

**Affiliations:** a Department of Obstetrics and Gynecology, Union Hospital, Tongji Medical College, Huazhong University of Science and Technology, Wuhan, China; b The First College of Clinical Medical Science, China Three Gorges University, Yichang, Hubei, China; c Department of Clinical Laboratory, Union Hospital, Tongji Medical College, Huazhong University of Science and Technology, Wuhan, China.

**Keywords:** EOC, miR-126, PFI, prognostic, survival

## Abstract

Ovarian cancer is the leading cause of gynecological cancer related death in females worldwide. Our previous study demonstrated that decreased expression of microRNA (miR-126) promoted ovarian cancer angiogenesis and invasion by targeting VEGF-A. This study aimed to evaluate the clinical validity of miR-126 as a prognostic marker for epithelial ovarian cancer (EOC).

**Patient concerns:**

The patients with EOC ranged in age from 27 to 79 years, with a mean age of 57 years.

**Diagnosis:**

All patients had never had chemotherapy or biotherapy, and the diagnoses were confirmed pathologically in all cases

**Methods:**

MiR-126 levels in EOC tissue and normal ovaries were determined by qRT-PCR. Its prognostic value was analyzed using the Cox proportional hazards regression model. Survival curves were drawn using the Kaplan–Meier method.

**Results:**

In this study, we found that compared to normal tissues, miR-126 expression was lower in EOC tissues, particularly in omental metastases. Though in our previous study we found that miR-126 may inhibit proliferation and invasion in EOC cell lines, but in this study patients with elevated miR-126 expression exhibited poor overall survival and relapse free survival. Multivariate Cox regression analysis showed that miRNA-126 was an independent prognostic factor for poor relapse-free survival (*P* = .044). Receiver operating characteristic analysis showed that the area under the curve of miR-126 was 0.806 (95% confidence interval, 0.669-0.942).

**Conclusion:**

In this study, we established miR-126 as a potential independent biomarker for predicting recurrence in patients with EOC.

## 1. Introduction

Despite tremendous improvements in treatment strategies, epithelial ovarian cancer (EOC) remains to be the first leading cause of gynecological cancer death in the world.^[1]^ 5-year survival rates are only 40% in most countries.^[2]^The ovarian cancer mortality rate ranks first among tumors of female reproductive system. It is estimated that 19,880 new cases of ovarian cancer will be diagnosed and 12,810 deaths due to ovarian cancer will occur in 2022 in United States.^[3]^ Even though the platinum-free interval (PFI) is considered as a major prognostic factor in guiding treatment selection in relapses,^[4]^ it is urgently needed to find a targeting marker in clinic to optimize the current diagnosis and treatment of ovarian cancer.

MicroRNAs are closely related to tumorigenesis and can act as oncogenes or tumor suppressor genes to influence the occurrence and development of tumor. Differentially expressed miRNA may be used as the indicator of early diagnosis, molecular typing and prognosis, including ovarian cancer.^[5–7]^ microRNA (miR-126), originally identified as an endothelial-specific miRNA playing an essential role in angiogenesis and vascular integrity,^[8,9]^ it has been shown to function as a critical tumor suppressor in various types of solid tumors.^[10–13]^

Overexpressed miR-126 can inhibit drug resistance cell proliferation and promote apoptosis of drug resistance cell, and invasion of cancer cells in various types of solid tumors.^[14]^ However, so far, the significance of miR-126 in the prognosis of ovarian cancer remains largely unclear. In this study, we identified miR-126 expression differences in 69 patients with primary EOC and 8 corresponding omental metastases. Our previous studies have indicated that the loss of expression of miR-126 contributes to the abnormal VEGF-A accumulation and subsequent unchecked cell invasion and cell proliferation in epithelial ovarian cancer.^[15]^ Interesting, in this study, we found that miR-126 was upregulated in the patients with resistant or refractory relapse (PFI < 6 months) compare to patients with partially sensitive or sensitive relapse (PFI > 6 months). We have indicated the potential of miR-126 to predict the risk of EOC recurrence. Our results demonstrate that miR-126 was an independent prognostic indicator in EOC.

## 2. Materials and methods

### 2.1. Tissue samples

Tumor and normal specimens were obtained from the Gynecologic Tissue Bank of Wuhan Union Hospital with written informed consent and ethical approval from the ethical committee of the hospital. No patients had been subjected to previous chemotherapy. All diagnoses were pathologically confirmed.

### 2.2. Patients and samples

Samples of human ovarian tissues were obtained from patients who underwent surgery at Wuhan Union Hospital from 2004 through 2019.In total, 23 normal ovarian tissues (germinal epithelium from patients with adenomyosis or myoma), 15 benign cystadenomas (5 serous cystadenomas and 10 mucinous cystadenomas), 8 borderline cystadenomas (4 mucinous cystadenomas and 4 serous cystadenomas) and 69 malignant epithelial tumors (1 undifferentiated tumor, 4 clear cell tumor, 3 endometrioid carcinomas, 11 mucinous cystadenocarcinomas and 50 serous cystadenocarcinomas) were analyzed. A total of 69 EOC patients (age 57 years, range 27–79 years).

### 2.3. Clinical follow up

Clinical follow up was available for 50 patients (median, 20 months; range, 2–75 months). Mortality occurred in 26 patients during the follow up period. Among those 50 *patients*, no recurrence was found in 18 cases. Overall survival (OS) and relapse-free survival (RFS) time were measured from the date of the initial surgery to death and relapse, respectively.

### 2.4. RNA extraction and real-time PCR analysis

Total RNA was extracted with Trizol reagent (Invitrogen, Carlsbad, CA) according to the supplied protocol. miR-126 was amplified in triplicate using a Hairpin-it™ microRNAs qPCR Quantitation Kit (GenePharma, Shang-Hai, China) on a Step One Plus system (Applied Biosystems, Foster City, CA) following the manufacturer’s instructions. Levels of gene expression were normalized to U6. The expression level of each gene was calculated using the 2^-∆∆CT^ method.

### 2.5. Statistical analysis

The comparisons of means were assessed using independent-samples and paired-samples t tests. The median expression value of miR-126 in the EOC tissues was used to classify high and low miR-126 expression groups. The Fisher exact test was used to evaluate the association of miR-126 expression with EOC clinicopathological characteristics. The OS and RFS were analyzed by Kaplan–Meier survival curve and the log–rank test. Hazard ratios and 95% confidence intervals for miR-126 expression were calculated from univariate and multivariate Cox regression model. A receiver operating characteristic (ROC) curve was plotted to evaluate the validity of miR-126 levels to distinguish patients with resistant or refractory relapse (PFI < 6 months) from patients with partially sensitive or sensitive relapse (PFI > 6 months). Statistical analyses were performed using SPSS 13.0 software (SPSS, Inc, Chicago, IL). *P* < .05 suggest a statistically significant different.

## 3. Results

### 3.1. miR-126 is down-regulated in epithelial ovarian cancer

**Figure 1.**
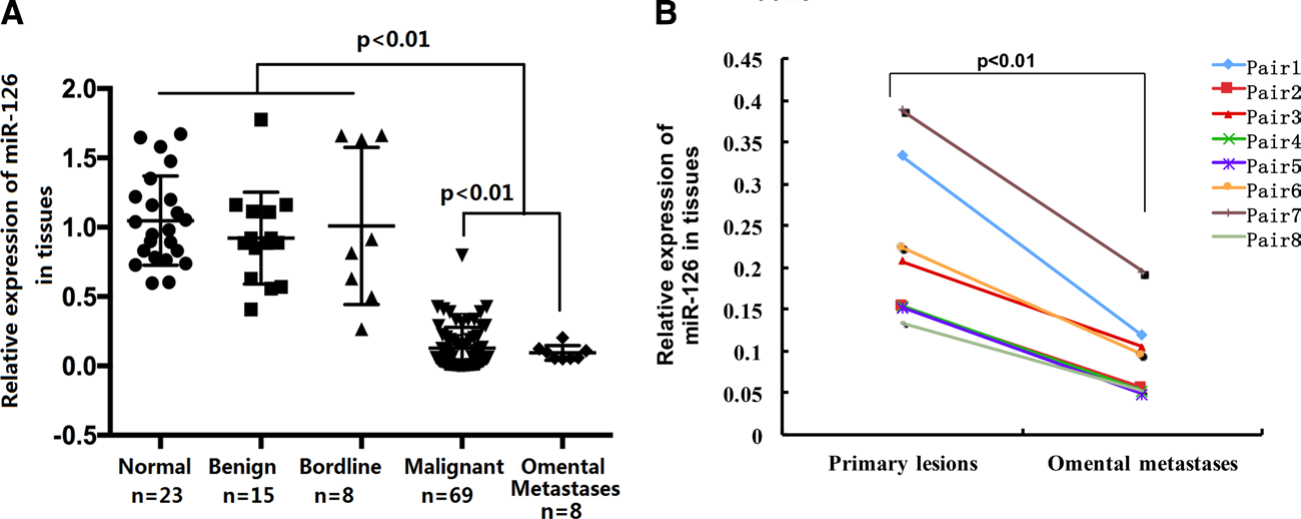
Differential expression of miR-126. (A) Relative expression of miR-126 in normal, benigh, bordline, malignant ovarian tissues and omental metastases was measured by quantitative real-time polymerase chain reaction (qRT-PCR) using *U6* as the reference gene. Results represent the means of the values. All reactions were run in triplicate. Relative quantification was performed using the 2^–ΔΔCT^ method. B, MiR-126 qRT-PCR in paired primary tumors and omental metastases. *U6* was the reference gene (n = 8 pairs). miR-126 = microRNA.

The expression levels of miR-126 in 115 clinical samples were assessed by qRT-PCR. The results revealed miR-126 levels were significantly lower in primary ovarian lesions and omental metastases than in the normal ovarian epithelium, benign lesion, and borderline tumor (shown in Fig. 1A). In the matched pairs (n = 8), all omental metastases had lower miR-126 expression than their corresponding primary tumors, suggesting that miR-126 may play a role in metastasis (shown in Fig. 1B).

In this study, no significant correlation was found for miR-126 with patients age, tumor stage, federation international of gynecology and obstetrics stage, histologic subtypes (shown in Table 1)

**Table 1:** Association between miR-126 expression and clinicopathological features of EOC.

Clinicopathological features	No. of cases	miR-126 expression No.%		
		Low	High	*P* value*
Age at surgery (yr)				
<45	25	14 (56.0)	11 (44.0)	.306
≥45	44	19 (43.2)	25 (56.8)	
FIGO stage				
I + II	30	14 (46.7)	16 (53.3)	.866
III + IV	39	19 (48.7)	20 (51.3)	
Tumor grade				
Low	30	16 (53.3)	14 (46.7)	.422
High	39	17 (43.6)	22 (56.4)	
Histological subtypes				
Serous	50	25 (50.0)	25 (50.0)	.558
Others	19	8 (42.1)	11 (57.9)	

EOC = epithelial ovarian cancer, FIGO = international federation of gynecology and obstetrics, miR-126 = microRNA.

* Fisher exact test.

### 3.2. miR-126 served as an independent prognostic factor for poor survival in EOC

**Figure 2.**
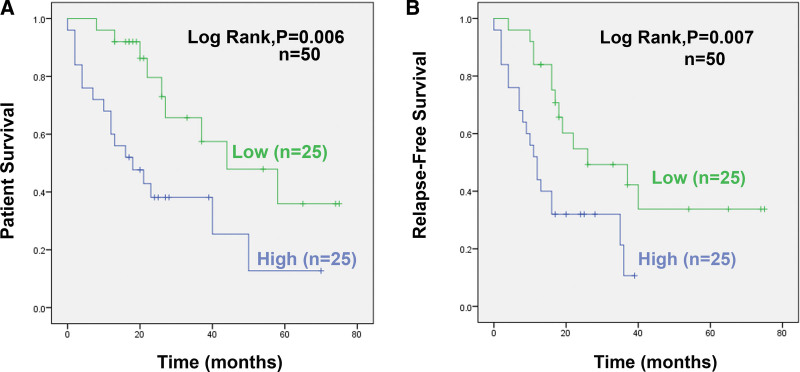
Kaplan–Meier survival curves for patients with 50 ovarian cancer based on the expression of miR-126. (A) Overall survival. (B) Relapse-free survival. The log-rank test gave significant *P* values (*P < *.05): A, *P = *.006 (n = 50); B, *P = *.007(n = 50). miR-126 = microRNA.

We next examined the relation between miR-126 expression and patient survival. Our data showed that EOC patients with higher miR-126 expression had significantly shorter Overall survival (*P* < .01) and relapse-free survival (*P* < .01) (shown in Fig. 2A and B). In the univariate analysis, federation international of gynecology and obstetrics stage, tumor grade and miR-126 expression significantly impacted RFS and OS rates (*P* < .05) (shown in Table 2). In multivariate analysis, however, only miR-126 expression remained significant, suggesting that miR-126 expression was an independent prognostic indicator for RFS (hazard ratios = 2.383; 95% confidence interval = 1.025-5.545; *P* = .044) (shown in Table 3).

**Table 2:** Univariate analysis of miR-126 expression and clinicopathological parameters in 50 patients with epithelial ovarian cancer.

Variables	Case (%)	OS		RFS	
		HR (95%CI)	*P* value	HR (95%CI)	*P* value
Age (yr)					
<45	18 (36)	0.815 (0.362–1.835)		0.931 (0.453–1.915)	
≥45	32 (64)	1.0	.621	1.0	.846
FIGO stage					
I–II	22 (44)	0.208 (0.075–0.575)		0.398 (0.187–0.849)	
III–IV	28 (56)	1.0	.002	1.0	.017
Cell differentiation					
Low	23 (46)	0.251 (0.102–0.615)		0.410 (0.198–0.848)	
High	27 (54)	1.0	.003	1.0	.016
Histological subtypes					
Serous	37 (74)	0.777 (0.323–1.868)		0.899 (0.401–2.018)	
Others	13 (26)	1.0	.573	1.0	.797
miR-126 expression*					
High	25 (50)	2.939 (1.297–6.661)		2.679 (1.284–5.588)	
Low	25 (50)	1.0	.010	1.0	.009

CI = confidence interval, HR = hazard ratio, OS = overall survival, RFS = relapse-free survival.

* The median expression level of miR-126 in the primary tumors was used as the cutoff value to categorize them into high and low expression groups.

**Table 3:** Cox multivariate analyses of prognostic factors on overall survival and relapse-free survival in patients with epithelial ovarian cancer.

Variables	Case (%)	COX (OS)		COX (RFS)	
		HR (95% CI)	*P* value	HR (95% CI)	*P* value
Age (yr)					
<45	18 (36)	1.149 (0.466–2.837)		1.229 (0.565–2.670)	
≥45	32 (64)	1.0	.763	1.0	.603
FIGO stage					
I–II	22 (44)	0.272 (0.070–1.050)		0.450 (0.167–1.213)	
III–IV	28 (56)	1.0	.059	1.0	.114
Cell differentiation					
Low	23 (46)	0.634 (0.176–2.285)		0.816 (0.300–2.217)	
High	27 (54)	1.0	.486	1.0	.690
Histological subtypes					
Serous	37 (74)	0.448 (0.170–1.181)		0.607 (0.256–1.439)	
Others	13 (26)	1.0	.104	1.0	.257
miR-126 expression					
High	25 (50)	2.421 (0.939–6.240)		2.383 (1.025–5.545)	
Low	25 (50)	1.0	.067	1.0	.044

CI = confidence interval, FIGO = federation international of gynecology and obstetrics, HR = hazard ratio, miR-126 = microRNA, OS = overall survival, RFS = relapse-free survival.

### 3.3. miR-126 could be the prognosis model for predicting the EOC Recurrence

**Figure 3.**
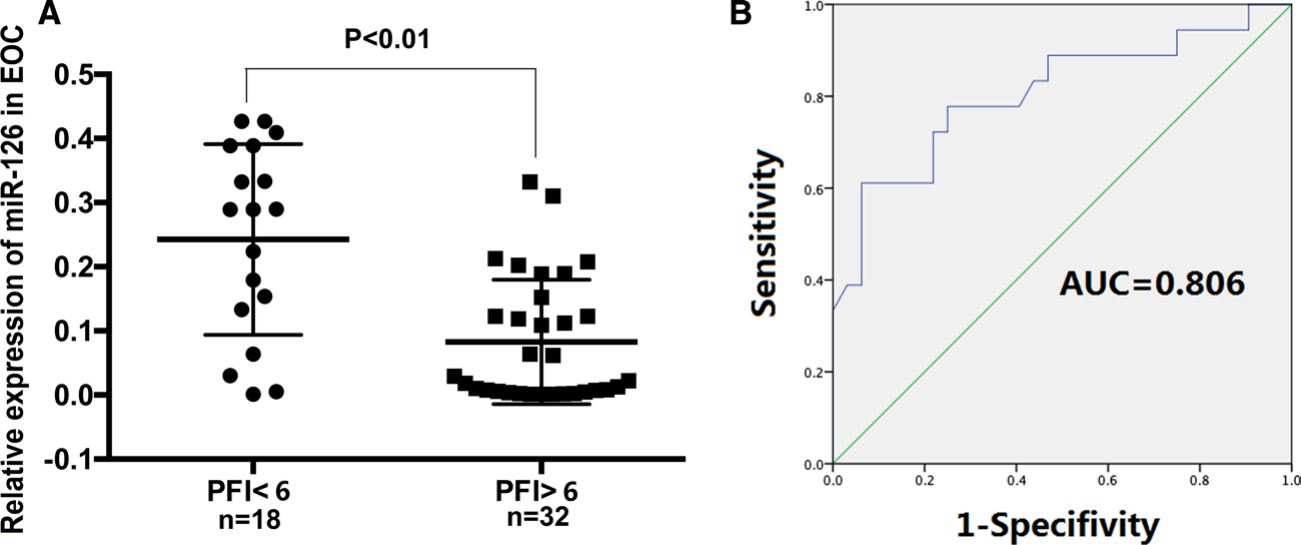
The expression level of miR-126 in epithelial ovarian cancer (EOC) samples with <6 months of PFI and with more than 6 months of PFI. (A) Relative expression of miR-126 in patients with <6 months of PFI and patients with more than 6 months of PFI was measured by quantitative real-time polymerase chain reaction using *U6* as the reference gene. Results represent the means of the values. All reactions were run in triplicate. Relative quantification was performed using the 2^–ΔΔCT^ method. (B) Receiver operating characteristic curves of miR-126 in classifying EOC samples with <6 months of PFI and with more than 6 months of PFI. Area under the curve = 0.806. miR-126 = microRNA, PFI = platinum-free interval.

Since miR-126 was a significant independent prognostic factor for RFS, we reexamined our data. To find out the prognostic significance of miR-126 in patients with EOC, the fold change in miR-126 level between patients with resistant or refractory relapse (PFI < 6 months) to patients with partially sensitive or sensitive relapse (PFI > 6 months) was analyzed and a ROC curve was established. miR-126 level in the patients with <6 months of PFI recurrences was increased when compared to the patients with more than 6 months of PFI (*P* < .05) (shown in Fig. 3A).

In ROC analysis miR-126 levels yielded an area under the ROC curve value of 0.806 (95% confidence interval: 0.666–0.940). The sensitivity was 77.8% and the specificity was 75%.at the cutoff value of 0.128, which represented highly predictive of tumor recurrence (shown in Fig. 3B).

## 4. Discussion

miR-126 is an intragenic miRNA. It is located on human chromosome 9, within the 7th intron of EGFL7.^[8]^ The function of miR-126 in physiological and pathological processes, including inflammation,^[16]^ blood vessel growth, and cancer,^[8]^ has been intensively studied. miR-126 is not only expressed in capillaries and larger vascular endothelial cells, but also highly expressed in highly vascularized tissues such as heart, liver and umbilical vein endothelial cells, and some studies have shown that it is expressed in progenitor cells, endothelial cells Lineage, expression in hematopoietic cell lines.^[9,17,18]^

miR-126 is down-regulated in most of the cancers studied. Aberrant expression of miR-126 is associated with the lung, thyroid, breast, genital tracts, gastrointestinal tract, and some other cancers in the previous studies. In cancer, miR-126 can inhibit the growth of tumor cells, metastasis, by inhibiting a series of important genetic targets (such as PI3K, KRAS, EGFL7, CRK, ADAM9, HOXA9, IRS-1, SOX-2, SLC7A5, and VEGF).^[8,15,19,20]^ Functions such as death, invasion and metastasis. In some cases, miR-126 promotes cancer progression by promoting blood vessel formation. Understanding these mechanisms of miR-126 involvement in cancer pathogenesis will help develop therapeutic targets for treating cancer patients.

In ovarian cancer, previous studies show that miR-126 is down-regulated in ovarian cancer cells and can inhibit cancer cell growth, migration, and invasion by suppressing a range of gene targets such as PAK4, VEGF, PLXNB2, in vitro.^[21]^ Furthermore, miR-126 is found to be underrepresented in plasma exosomes from patients with EOC compared to healthy women.^[22]^ In concordance with this, we found that miR-126 was underrepresented in ovarian cancer (OC) samples. However, Resnick et al^[23]^ also observed a contradicting finding that miR-126 was overexpressed in sera from patients with OC. However, elevated serum levels of miRNA may not necessarily mean that these microRNAs are produced by cancers themselves.

In concordance with another study,^[24]^ we found that higher miR-126 levels was associated the shorter overall survival and elevated miR-126 levels was an independent predicative factor for EOC Recurrence. This finding may seem to be contradictory to the observation that miR-126 was down-regulated in OC specimens. However, a recent study shows that miR-126 confers platinum resistance in epithelial ovarian cancer through the activation of β-catenin/CBP signaling by targeting its negative regulators such as DKK3, AXIN1, BACH1, and NFAT5.^[25]^ Considering platinum resistant relapses account for most of the mortality, these findings may offer an explanation. That is, in the last stage, the development of chemoresistance may play a more important role in the mortality. However, whether this is the case, needs further studies.

In addition, because our sample for the study came from only 1 medical center, our findings may not apply to all EOC populations. However, our research is consistent with existing published articles, and there may not be major deviations.

In summary, in our study, we described the differential expression of miR-126 in the EOC patient cohort and demonstrate the potential utility of miR-126 as a prognostic marker in EOC.As far as we know, it is the first time we demonstrated that high expression of miR-126 was independent prognostic factors for OS and RFS. Therefore, miR-126 may serve as a molecular marker for early detection, postoperative monitoring recurrence, even targeted therapy for epithelial ovarian cancer in the future.

## Author contributions

**Conceptualization:** Jing Cai, Da Huang.

**Data curation:** Lin Liu, Qing Han, Man Xiao, Jin Cao.

**Formal analysis:** Lin Liu, Qing Han, Jing Cai, Jin Cao.

**Investigation:** Lin Liu, Jing Cai, Man Xiao.

**Methodology:** Lin Liu, Qing Han, Jing Cai, Man Xiao, Jin Cao.

**Project administration:** Qing Han, Da Huang, Jin Cao.

**Supervision:** Lin Liu, Jin Cao.

**Validation:** Jin Cao.

**Writing – original draft:** Lin Liu, Qing Han, Da Huang, Jin Cao.

**Writing – review & editing:** Lin Liu, Qing Han, Jin Cao.

## References

[1] Armstrong DK Alvarez RD Bakkum-Gamez JN. Ovarian cancer, version 2.2020, NCCN clinical practice guidelines in oncology. J Natl Compr Canc Netw. 2021;19:191–226. 33545690 10.6004/jnccn.2021.0007

[2] Gardner AB Charo LM Mann AK. Ovarian, uterine, and cervical cancer patients with distant metastases at diagnosis: most common locations and outcomes. Clin Exp Metastasis. 2020;37:107–13. 31758289 10.1007/s10585-019-10007-0

[3] Siegel RL Miller KD Fuchs HE. Cancer statistics, 2022. CA Cancer J Clin. 2022;72:7–33. 35020204 10.3322/caac.21708

[4] Pignata S S CC Du Bois A. Treatment of recurrent ovarian cancer. Ann Oncol. 2017;28(suppl_8):viii51–6. 29232464 10.1093/annonc/mdx441

[5] Xu C Xie J Liu Y. MicroRNA expression profiling and target gene analysis in gastric cancer. Medicine (Baltim). 2020;99:e21963. 10.1097/MD.0000000000021963 PMC7489646 32925730

[6] Chen Z Xiao Z Zeng S. The potential value of microRNA-145 for predicting prognosis in patients with ovarian cancer: a protocol for systematic review and meta-analysis. Medicine (Baltim). 2021;100:e26922. 10.1097/MD.0000000000026922 PMC8360411 34397934

[7] Liu B Pan J Fu C. Correlation of microRNA-367 in the clinicopathologic features and prognosis of breast cancer patients. Medicine (Baltim). 2021;100:e26103. 10.1097/MD.0000000000026103 PMC8183767 34087856

[8] Yang QY Yu Q Zeng WY. Killing two birds with one stone: miR-126 involvement in both cancer and atherosclerosis. Eur Rev Med Pharmacol Sci. 2022;26:6145–68. 36111944 10.26355/eurrev_202209_29632

[9] Sufianov A Begliarzade S Kudriashov V. Role of miRNAs in vascular development. Noncoding RNA Res. 2023;8:1–7. 36262425 10.1016/j.ncrna.2022.09.010 PMC9552023

[10] Li Q Wang G Wang H. miR-126 functions as a tumor suppressor by targeting SRPK1 in human gastric cancer. Oncol Res. 2018;26:1345–53. 29510776 10.3727/096504018X15180508535835 PMC7844751

[11] Sibilano M Tullio V Adorno G. Platelet-derived miR-126-3p directly targets AKT2 and exerts anti-tumor effects in breast cancer cells: further insights in platelet-cancer interplay. Int J Mol Sci. 2022;23:5484. 35628294 10.3390/ijms23105484 PMC9141257

[12] Chen CM Chu TH Chou CC. Exosome-derived microRNAs in oral squamous cell carcinomas impact disease prognosis. Oral Oncol. 2021;120:105402. 34174519 10.1016/j.oraloncology.2021.105402

[13] Selven H Busund LR Andersen S. High expression of microRNA-126 relates to favorable prognosis for colon cancer patients. Sci Rep. 2021;11:9592. 33953222 10.1038/s41598-021-87985-3 PMC8100289

[14] Heissig B Salama Y Takahashi S. The multifaceted roles of EGFL7 in cancer and drug resistance. Cancers (Basel). 2021;13:1014. 33804387 10.3390/cancers13051014 PMC7957479

[15] Liu L Yuan L Huang D. miR126 regulates the progression of epithelial ovarian cancer in vitro and in vivo by targeting VEGFA. Int J Oncol. 2020;57:825–34. 32705156 10.3892/ijo.2020.5082

[16] Ginckels P Holvoet P. Oxidative stress and inflammation in cardiovascular diseases and cancer: role of non-coding RNAs. Yale J Biol Med. 2022;95:129–52. 35370493 PMC8961704

[17] Wang H Sun J Zhang B. Targeting miR-126 disrupts maintenance of myelodysplastic syndrome stem and progenitor cells. Clin Transl Med. 2021;11:e610. 34709739 10.1002/ctm2.610 PMC8516361

[18] Hoang DH Zhao D Branciamore S. MicroRNA networks in FLT3-ITD acute myeloid leukemia. Proc Natl Acad Sci U S A. 2022;119:e2112482119. 35412895 10.1073/pnas.2112482119 PMC9169767

[19] Bi X Lv X Liu D. METTL3-mediated maturation of miR-126-5p promotes ovarian cancer progression via PTEN-mediated PI3K/Akt/mTOR pathway. Cancer Gene Ther. 2021;28:335–49. 32939058 10.1038/s41417-020-00222-3

[20] Li Y Li Y Ge P. Correction to: MiR-126 regulates the ERK pathway via targeting KRAS to inhibit the glioma cell proliferation and invasion. Mol Neurobiol. 2021;58:1872. 33496908 10.1007/s12035-021-02302-3

[21] Xiang G Cheng Y. MiR-126-3p inhibits ovarian cancer proliferation and invasion via targeting PLXNB2. Reprod Biol. 2018;18:218–24. 30054097 10.1016/j.repbio.2018.07.005

[22] Pan C Stevic I Muller V. Exosomal microRNAs as tumor markers in epithelial ovarian cancer. Mol Oncol. 2018;12:1935–48. 30107086 10.1002/1878-0261.12371 PMC6210043

[23] Resnick KE Alder H Hagan JP. The detection of differentially expressed microRNAs from the serum of ovarian cancer patients using a novel real-time PCR platform. Gynecol Oncol. 2009;112:55–9. 18954897 10.1016/j.ygyno.2008.08.036

[24] Prahm KP Hogdall C Karlsen MA. Identification and validation of potential prognostic and predictive miRNAs of epithelial ovarian cancer. PLoS One. 2018;13:e0207319. 30475821 10.1371/journal.pone.0207319 PMC6261038

[25] Wu G Cao L Zhu J. Loss of RBMS3 confers platinum resistance in epithelial ovarian cancer via activation of miR-126-5p/beta-catenin/CBP signaling. Clin Cancer Res. 2019;25:1022–35. 30279231 10.1158/1078-0432.CCR-18-2554

